# Increase of PD-L1 expressing B-precursor ALL cells in a patient resistant to the CD19/CD3-bispecific T cell engager antibody blinatumomab

**DOI:** 10.1186/s13045-015-0213-6

**Published:** 2015-10-08

**Authors:** Thomas Köhnke, Christina Krupka, Johanna Tischer, Thomas Knösel, Marion Subklewe

**Affiliations:** Department of Internal Medicine III, Ludwig-Maximilians-Universität (LMU), Munich, Germany; Clinical Cooperation Group Immunotherapy at the Helmholtz Zentrum München, Munich, Germany; Institute of Pathology, Ludwig-Maximilians-Universität (LMU), Munich, Germany

**Keywords:** ALL, Immunotherapy, Blinatumomab, Immune checkpoints, T cells, Combination therapy

## Abstract

The bispecific T cell engager blinatumomab has shown encouraging clinical activity in B-precursor acute lymphoblastic leukemia (ALL). However, about half of relapsed/refractory patients do not respond to therapy. Here, we present the case of a 32-year-old male patient with refractory B-precursor ALL who was resistant to treatment with blinatumomab. Bone marrow immunohistochemistry revealed T cell infiltrates and an increase in programmed death-ligand 1 (PD-L1)-positive ALL cells as a potential immune escape mechanism. We were able to recapitulate the clinical observation *in vitro* by showing that blinatumomab was not able to mediate cytotoxicity of CD19-positive ALL cells using autologous T cells. In contrast, the addition of healthy donor T cells led to lysis of ALL cells.

These results strongly encourage further systematic evaluation of checkpoint molecules in cases of blinatumomab treatment failure and might highlight a possible mechanism to overcome resistance to this otherwise highly effective treatment.

## Background

Bispecific T cell engaging (BiTE®) antibody constructs represent a novel class of therapeutic antibodies, which are comprised of two single-chain variable fragments simultaneously binding CD3-positive T cells and a specific tumor target antigen [1]. Blinatumomab is the first of BiTE® antibody construct which entered the clinic and simultaneously binds CD3-positive T cells and CD19-positive B cells. The design of this BiTE® molecule brings CD19-positive target cells in close contact with CD3-positive T cells. Importantly, binding of the BiTE® molecule results in polyclonal T cell activation and expansion which results in effective lysis of the target cells irrespective of T cell specificity [2, 3]. Blinatumomab has shown to have antileukemic activity in patients with B-precursor acute lymphoblastic leukemia (ALL) [4], and recently, blinatumomab was approved for the treatment of relapsed or refractory B-precursor ALL by the FDA (http://www.fda.gov/drugs/informationondrugs/approveddrugs/ucm425597.htm). Loss of CD19 and extramedullary relapse have been observed as mechanisms of resistance to blinatumomab treatment [5, 6]; however, other mechanisms of resistance have not been reported so far.

Upregulation of programmed death-ligand 1 (PD-L1) on tumor cells in response to endogenous anti-tumor immunity [7] inhibits adaptive immune responses by inducing T cell dysfunction [8]. Expression of PD-L1 on tumor cells has been associated with poor outcome in solid cancers [9] as well as hematologic malignancies [10]. Antibodies targeting PD-L1 as well as its receptor on T cells, programmed cell death-1 (PD-1), are being evaluated in a variety of cancers [9, 11, 12], including lymphoid malignancies [7]. Recently, two antibodies targeting PD-1 were granted approval for the treatment of advanced melanoma (pembrolizumab, nivolumab) as well as metastatic squamous non-small cell lung cancer (nivolumab). Interestingly, activity of these therapies is not limited to PD-L1-positive cancers, as clinical responses could be detected in cases with low PD-L1 expression [13]. There is extensive research in the field trying to develop improved predictive biomarkers to identify those patients which will response to mono- as well as combination immunotherapies [14].

### Case presentation

**Fig. 1:**
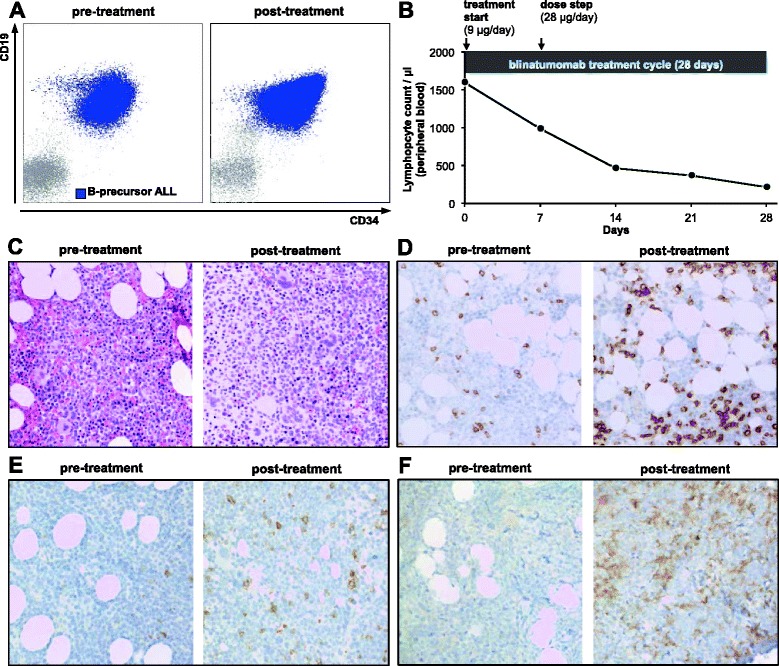
Increase of PD-1 and PD-L1 positivity after treatment with blinatumomab. **a** CD19 vs. CD34 expression of lymphoblasts detected by flow cytometry. Lymphoblasts showed homogenous expression of CD19 at baseline (pre-treatment) as well as after blinatumomab treatment (post-treatment). **b** Lymphocyte counts on peripheral blood during blinatumomab treatment. Lymphocyte counts decreased during blinatumomab treatment (1596/μl on day 0, 986/μl on day 7, 464/μl on day 14, 368/μl on day 21, and 217/μl on day 28). **c** Hematoxylin and eosin stain of paraffin embedded bone marrow core biopsy showing diffuse infiltration of immature progenitors at both time points (pre-treatment blast count 30 %, post-treatment 60 %). **d** Immunohistochemistry of paraffin embedded bone marrow core biopsy stained for CD3 showing spotted infiltration of CD3-positive T cells at baseline (5–10 %, pre-treatment) and diffuse infiltration after blinatumomab treatment (20–30 %, post-treatment). **e** Immunohistochemistry of paraffin embedded bone marrow core biopsy stained for PD-1 showing 5 % PD-1-positive cells at baseline (pre-treatment) vs. 15 % after blinatumomab treatment (post-treatment). **f** Immunohistochemistry of paraffin embedded bone marrow core biopsy stained for PD-L1 showing 2 % PD-L1-positive blasts at baseline (pre-treatment) vs. 40 % after blinatumomab treatment (post-treatment)

A 32-year-old male patient presented with refractory B-precursor ALL after frontline treatment with induction I and II as well as consolidation I of the German multicenter study group on adult acute lymphoblastic leukemia (GMALL) treatment protocol (analogous to the GMALL trial 07/2003, *Clinicaltrials.gov identifier: 00198991*). He was subsequently transferred to our center and received blinatumomab as a continuous infusion at a dose of 9 μg/day for 7 days and 28 μg/day for the subsequent 21 days. Bone marrow blast count prior to treatment with blinatumomab (baseline) was 30 % (Fig. 1c, left panel), and lymphoblasts homogenously expressed CD19 (Fig. 1a, left panel). Treatment was well tolerated with pyrexia on days 1 through 4 of blinatumomab treatment being the only adverse event. Examination of peripheral blood revealed a decrease of total lymphocyte counts during treatment (1596/μl on day 0 vs. 217/μl on day 28, Fig. 1b) including decreased T cells (960 CD3-positive T cells/μl on day 5 vs. 180/μl on day 28). Interestingly, there was a moderate increase in CD3-positive T cells within the bone marrow (5–10 % at baseline vs. 20–30 % after blinatumomab treatment, Fig. 1d).

**Fig. 2:**
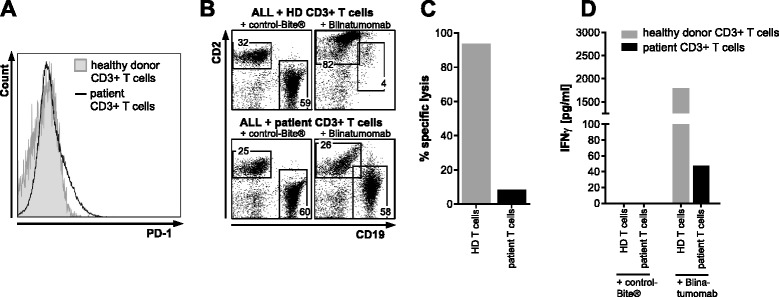
Decreased *in vitro* blinatumomab-mediated lysis of ALL blasts by patient CD3-positive T cells. **a** PD-1 expression on peripheral CD3-positive T cells was compared to PD-1 expression on healthy donor CD3-positive T cells showing no detectable difference. **b** The patient’s ALL blasts were cocultured either with healthy donor CD3-positive T cells (*upper panels*) or the patient’s own CD3-positive T cells (*lower panels*) with either control-Bite® (*left panels*) or blinatumomab (*right panels*) and analyzed by flow cytometry after 3 days. **c** Specific lysis was calculated as one minus the ratio of CD19-positive cells treated with blinatumomab and CD19-positive cells treated with control-Bite®. Healthy donor CD3-positive T cells showed efficient lysis of our patient’s ALL blasts (specific lysis 93.6 %) whereas CD3-positive T cells from our patient showed inefficient lysis of autologous ALL blasts (specific lysis 8.5 %). **d** IFN-γ concentration in cell culture supernatants were considerably lower for our patient’s T cells cocultured with his ALL blasts, whereas coculture of healthy donor T cells and ALL blasts from our patient led to considerable IFN-γ production. Cell cultures with control-Bite® did not show any IFN-γ production

Upon completion of the first cycle, bone marrow examination revealed persistent leukemia with a blast count of 60 % (Fig. 1c, right panel), showing homogenous expression of CD19 (Fig. 1a, right panel). Since a loss of CD19 as the mechanism of resistance could not be detected, we examined PD-1 and PD-L1 expression in the bone marrow by immunohistochemistry. A moderate increase in PD-1 positivity was seen in lymphocytes in the bone marrow by immunohistochemistry (5 % PD-1-positive cells at baseline vs. 15 % after blinatumomab treatment, Fig. 1e). However, we observed a marked increase of PD-L1 positivity (2 % PD-L1-positive blasts at baseline vs. 40 % after blinatumomab treatment, Fig. 1f). In contrast to the bone marrow, no PD-1 expression was observed on T cells from the peripheral blood (Fig. 2a).

Finally, we collected the patient’s peripheral blood after blinatumomab treatment and purified CD3-positive T cells (EasySep™ Human CD3 Positive Selection Kit II, Stemcell Technologies, Vancouver, British Columbia, Canada). Of either patient or healthy donor CD3-positive T cells, 2.5 × 10^5^ were cocultured with 5 × 10^5^ of the patient’s ALL cells (CD3-negative cells) with blinatumomab or control-BiTE® at a dose of 5 ng/ml on 3 × 10^4^ irradiated MS-5 feeder cells. After 3 days, cells were stained with CD19-PE, CD2-BV421, and murine CD29-APC-Cy7 (all BioLegend, San Diego, CA, USA), as well as LIVE/DEAD® Fixable Aqua Dead Cell Stain Kit (Life Technologies, Carlsbad, CA, USA), and analyzed on a LSR II flow cytometer (BD Biosciences, Heidelberg, Germany). After cell counting, the percentage of the respective cell population determined by flow cytometry was used to determine the absolute cell counts. Percentage of specific lysis was calculated using cell counts of control-BiTE® antibody-treated relative to blinatumomab-treated cultures, as described previously [15]. The interferon-γ (IFN-γ) concentration in cell culture supernatants was measured by cytometric bead array (BD™ CBA Human IFN-γ Flex Set, BD Biosciences, Heidelberg, Germany) according to the manufacturer’s instructions on the same machine.

*In vitro*, the patient’s T cells showed considerably less lysis of CD19-positive cells (8.5 vs. 93.6 % for healthy donor T cells, Fig. 2b, c). This was accompanied by considerably lower concentrations of IFN-γ in cell culture supernatants (47.7 vs. 2100.9 pg/ml for healthy donor T cells, Fig. 2d). These results recapitulate the clinical experience with an inability of the patients’ T cells to perform blinatumomab-mediated lysis of ALL cells.

## Discussion and conclusions

Taken together, our data suggests a role of PD-L1 in treatment resistance to blinatumomab in our patient. This is, to our knowledge, the first report of increased PD-L1 positivity in a patient receiving blinatumomab and should encourage a systematic evaluation of the relevance of this resistance mechanism in patients receiving bispecific T cell engager therapy. A large body of evidence has been suggesting a key role of the PD-1/PD-L1 axis in attenuating anti-tumor immune responses [16, 17]. Recently, we described this mechanism of resistance in the context of BiTE® antibody immunotherapy *in vitro* [18]. Detecting an increase of PD-L1 in a patient receiving blinatumomab therefore highlights the relevance of this mechanism *in vivo*.

Additionally, several resistance mechanisms to antibody-based immunotherapy have been reported including variation of target antigen expression (which might be present initially or develop during therapy), activation of alternative signaling pathways, and anti-antibody formation [19–22]. In the context of novel immunotherapies, including blinatumomab, assessment of resistance mechanisms is limited and warrants further investigation. Upregulation of checkpoint molecules represents an adaptive resistance to anti-tumor immunity which we hypothesize will also take place in other T cell recruiting antibody formats (e.g., DARTS, diabodies) as well as adoptive T cell therapies (e.g., CAR T cells, tumor antigen-specific TCR T cells). It will be important to conduct close monitoring of biomarkers and careful consideration of specific time points recognizing the dynamic interplay of receptor-ligand interactions. Further studies are warranted to analyze the significance of the PD-1/PD-L1 interplay as a resistance mechanism to blinatumomab. Ultimately, combinatorial approaches might have the potential to revert T cell-induced immune escape strategies and avoid treatment failure in these otherwise highly effective treatments.

### Statement of informed consent

Written informed consent was obtained from the patient for publication of this manuscript. A copy of the written consent is available for review by the Editor-in-Chief of this journal.

## Abbreviations

ALL: acute lymphoblastic leukemia
CAR T cells: chimeric antigen receptor T cells
DARTs: dual affinity re-targeting molecules
GMALL: German multicenter study group on adult acute lymphoblastic leukemia
IFN-γ: interferon-γ
PD-1: programmed cell death-1
PD-L1: programmed death-ligand 1
TCR: T cell receptor
